# Peer Review of “The Association of Shared Care Networks With 30-Day Heart Failure Excessive Hospital Readmissions: Longitudinal Observational Study”

**DOI:** 10.2196/37057

**Published:** 2022-04-06

**Authors:** Peng Zhao

**Affiliations:** 1 Institute for Data Science and Informatics University of Missouri Columbia, MO United States

**Keywords:** patient readmission, quality assurance, health care, catchment area, health, community networks, regional medical programs

*This is a peer-review report submitted for the paper “The Association of Shared Care Networks With 30-Day Heart Failure Excessive Hospital Readmissions: Longitudinal Observational Study.”*

## General Comments

Thank you for the opportunity to review this study [1] of the association of shared care networks with heart failure (HF) excessive hospital readmissions. Hospital readmission is a very current topic. Nonetheless, several issues should be noted.

## Specific Comments

### Major Comments

1. In “study population and design” in “methods,” the authors mentioned, “hospitals with less than 2 repeated measures of higher-than-expected HF readmission in the HRRP (Hospital Reduction Readmission Program) or without discharge data in the OSHPD (Office of Statewide Health Planning and Development) were excluded.” Does this mean this study only considered hospitals with repeated higher-than-expected HF readmission? Ignoring hospitals without repeated higher-than-expected HF readmission may introduce bias to the analysis. Please clarify why you have chosen this data inclusion criterion.
2. In “data sources” in “methods,” the authors collected excessive readmission ratio (ERR) data from 2012 to 2017. In almost every year, the HRRP updated the inclusion criteria of HF readmission (eg, lists of eligible diagnosis codes and procedure codes in the planned readmission algorithm). In this case, how did you fairly compare the ERR across different years?
3. Is the “Uncovering Shared Care Areas and Localization Index from Hospital-Patient Discharge Data” in “methods” a literature review of other studies or the method the authors used in this study? Please clarify. If it is a literature review, it should go in the “introduction.”

## Abbreviations

ERR: excessive readmission ratio
HF: heart failure
HRRP: Hospital Reduction Readmission Program
OSHPD: Office of Statewide Health Planning and Development
